# Predicting High Flow Nasal Cannula Failure in an Intensive Care Unit Using a Recurrent Neural Network With Transfer Learning and Input Data Perseveration: Retrospective Analysis

**DOI:** 10.2196/31760

**Published:** 2022-03-03

**Authors:** George Pappy, Melissa Aczon, Randall Wetzel, David Ledbetter

**Affiliations:** 1 The Laura P. and Leland K. Whittier Virtual PICU Children's Hospital Los Angeles Los Angeles, CA United States

**Keywords:** high flow nasal cannula, HFNC failure, predictive model, deep learning, transfer learning, LSTM, RNN, input data perseveration

## Abstract

**Background:**

High flow nasal cannula (HFNC) provides noninvasive respiratory support for children who are critically ill who may tolerate it more readily than other noninvasive ventilation (NIV) techniques such as bilevel positive airway pressure and continuous positive airway pressure. Moreover, HFNC may preclude the need for mechanical ventilation (intubation). Nevertheless, NIV or intubation may ultimately be necessary for certain patients. Timely prediction of HFNC failure can provide an indication for increasing respiratory support.

**Objective:**

The aim of this study is to develop and compare machine learning (ML) models to predict HFNC failure.

**Methods:**

A retrospective study was conducted using the Virtual Pediatric Intensive Care Unit database of electronic medical records of patients admitted to a tertiary pediatric intensive care unit between January 2010 and February 2020. Patients aged <19 years, without apnea, and receiving HFNC treatment were included. A long short-term memory (LSTM) model using 517 variables (vital signs, laboratory data, and other clinical parameters) was trained to generate a continuous prediction of HFNC failure, defined as escalation to NIV or intubation within 24 hours of HFNC initiation. For comparison, 7 other models were trained: a logistic regression (LR) using the same 517 variables, another LR using only 14 variables, and 5 additional LSTM-based models using the same 517 variables as the first LSTM model and incorporating additional ML techniques (transfer learning, input perseveration, and ensembling). Performance was assessed using the area under the receiver operating characteristic (AUROC) curve at various times following HFNC initiation. The sensitivity, specificity, and positive and negative predictive values of predictions at 2 hours after HFNC initiation were also evaluated. These metrics were also computed for a cohort with primarily respiratory diagnoses.

**Results:**

A total of 834 HFNC trials (455 [54.6%] training, 173 [20.7%] validation, and 206 [24.7%] test) met the inclusion criteria, of which 175 (21%; training: 103/455, 22.6%; validation: 30/173, 17.3%; test: 42/206, 20.4%) escalated to NIV or intubation. The LSTM models trained with transfer learning generally performed better than the LR models, with the best LSTM model achieving an AUROC of 0.78 versus 0.66 for the 14-variable LR and 0.71 for the 517-variable LR 2 hours after initiation. All models except for the 14-variable LR achieved higher AUROCs in the respiratory cohort than in the general intensive care unit population.

**Conclusions:**

ML models trained using electronic medical record data were able to identify children at risk of HFNC failure within 24 hours of initiation. LSTM models that incorporated transfer learning, input data perseveration, and ensembling showed improved performance compared with the LR and standard LSTM models.

## Introduction

### Background

The use of high flow nasal cannula (HFNC) respiratory support in children in critical care, emergency departments, and general wards has increased in recent years [1-8]. HFNC provides an alternative to other noninvasive ventilation (NIV) techniques and endotracheal intubation, has fewer associated risks and complications, and is well-tolerated by children [3,5,6,8]. Nevertheless, many patients require escalation to a higher level of respiratory support [3,8]. Importantly, for those who require escalation, recent research indicates better clinical outcomes for patients who were escalated to higher levels of respiratory support earlier: lower hospital and intensive care unit (ICU) mortality rates, higher extubation success rate, higher ventilator-free days, and lower hospital and ICU lengths of stay [9,10]. These findings suggest that early identification of children in whom HFNC will not be successful could allow more timely institutions of advanced respiratory support and decrease morbidity and mortality.

### Goals

This study aims to develop a model to make reliable, real-time predictions of a child’s response to HFNC. Such a model could help clinicians differentiate three groups: (1) children likely to do well on HFNC alone, (2) children likely to need a higher level of support, and (3) children whose HFNC response is unclear. Differentiating these 3 groups would help clinicians resolve the dilemma of appropriate NIV while not unduly and potentially harmfully prolonging it. The last group may benefit from the closest and most frequent monitoring. The second group, although still monitored frequently, could be escalated by clinicians to a higher level of support earlier. A further goal is to compare different algorithms, from logistic regressions (LRs) to long short-term memory (LSTM)-based recurrent neural networks, for predicting HFNC response. Other techniques, such as transfer learning (TL), input data perseveration, and ensembling, are also explored and evaluated for their impact on performance when used with LSTMs.

### Related Prior Work

The authors are unaware of any studies developing a predictive model of HFNC failure, although a few studies have investigated risk factors for escalation from HFNC to a higher level of respiratory support. Guillot et al [11] found that high pCO_2_ (partial pressure of carbon dioxide) was a risk factor for HFNC failure in children with bronchiolitis. Er et al [12] reported that respiratory acidosis, low initial oxygen saturation and SF (oxygen saturation [SpO_2_] divided by the fraction of inspired oxygen [FiO2]) ratio, and SF ratio <195 during the first few hours were associated with unresponsiveness to HFNC in children with severe bacterial pneumonia in a pediatric emergency department. In a small study of children with bacterial pneumonia, Yurtseven and Saz [13] saw higher failure rates in those with higher respiratory rates. Kelley et al [8] found that a high respiratory rate, high initial venous pCO_2_, and a pH <7.3 were associated with failure of HFNC.

## Methods

### Data Sources

Data for this study came from deidentified clinical observations collected in electronic medical records (EMRs; Cerner) of children admitted to the pediatric intensive care unit (PICU) of Children’s Hospital Los Angeles (CHLA) between January 2010 and February 2020. An episode represents a single admission and a contiguous stay in the PICU. Patients may have >1 episode. EMR data for an episode included irregularly, sparsely, and asynchronously charted physiological measurements (eg, heart rate and blood pressure), laboratory results (eg, creatinine and glucose level), drugs (eg, epinephrine and furosemide), and interventions (eg, intubation, bilevel positive airway pressure [BiPAP], or HFNC). Data previously collected for Virtual Pediatric Services, LLC participation [14], including diagnoses, gender, race, and disposition at discharge, were linked with the EMR data before deidentification.

### Ethics Exemption

The CHLA institutional review board reviewed the study protocol and waived the requirement for consent and institutional review board approval.

### Definitions

For ease of reference, Textbox 1 lists the terminologies and definitions used throughout the sections which follow.

**Useful definitions.**

**Terms and definitions**

- Episode: An individual child’s single, contiguous stay in the pediatric intensive care unit, spanning the time between admission and discharge
- High flow nasal cannula (HFNC) initiation: The start of HFNC treatment for a child not currently on HFNC
- HFNC period: The 24 hours following an HFNC initiation where the child was not on HFNC support at any time during the preceding 24 hours
- HFNC trial: An episode or subset of an episode (starting with admission) where only the very last HFNC period is designated as the training target; it may include previous HFNC initiations

**Figure 1:**
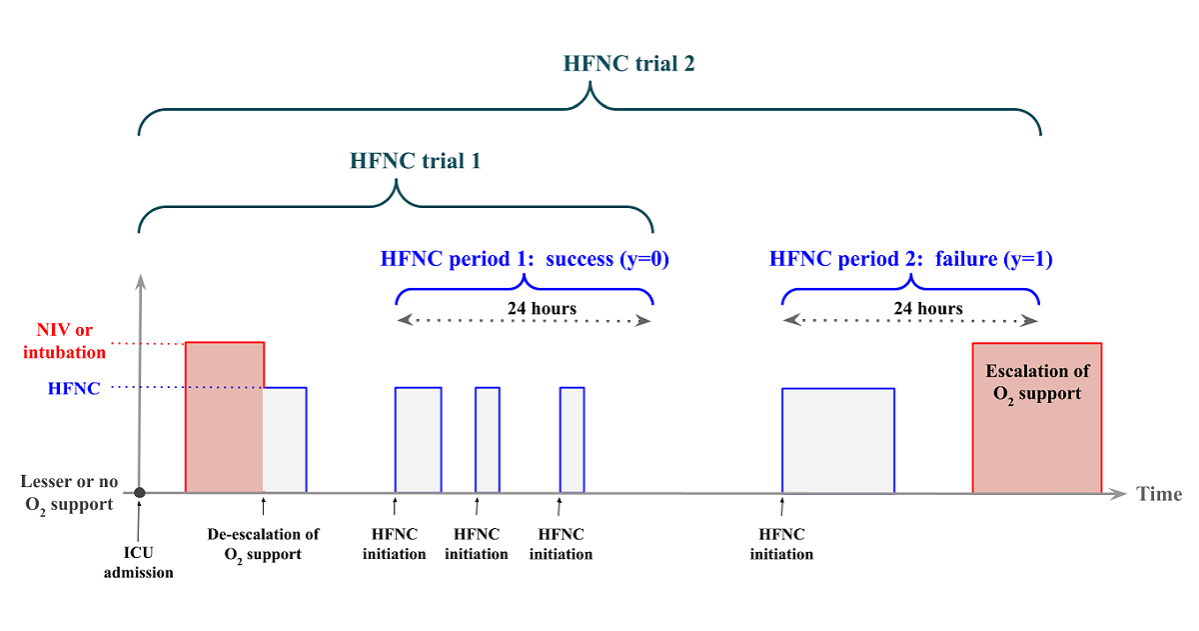
Illustration of HFNC scenarios, definitions, and outcomes. HFNC: high flow nasal cannula; ICU: intensive care unit; NIV: noninvasive ventilation.

In an episode where HFNC is initiated only once, there is exactly 1 HFNC period and 1 HFNC trial. Episodes can have multiple HFNC initiations. In such cases, a single episode may have multiple HFNC periods, and each has an associated HFNC trial. Note that not all HFNC initiations have a corresponding HFNC period. For instance, if a child started on HFNC for the first time during an episode, then this marked the start of the HFNC period. If HFNC was withdrawn 2 hours later, and the child again received HFNC an hour after that, then this new HFNC initiation did not mark the start of a new HFNC period as the original HFNC period had not yet ended. In contrast, if this second HFNC initiation took place >24 hours after the first HFNC was stopped, then this second initiation marked the start of a new HFNC period as it was initiated after the first HFNC period had already ended. Finally, at least 30 minutes was required between any de-escalation (*step-down*) from NIV or intubation before the start of the HFNC period. This rule was necessary as patients on a higher level of support may be stepped down to HFNC to assess their ability to breathe on their own. If such breathing trials fail, which is not an uncommon occurrence, these patients immediately escalate back to mechanical ventilation or NIV, technically becoming HFNC failures but were, in fact, extubation failures and are not representative of the escalation scenarios of interest in this study. Figure 1 illustrates these terminologies.

### Data Inclusions and Exclusions

Only episodes in which HFNC was used were included. Episodes of patients aged ≥19 years at admission were excluded, as were episodes associated with sleep apnea. Any episode that ended <24 hours into an HFNC period where the patient next went to the operating room was also excluded. Episodes with a do not intubate or do not resuscitate order were also excluded.

### Target Outcome

For each HFNC trial, the target of interest was escalation to a higher level of support (BiPAP, noninvasive mechanical ventilation, and intubation) within the 24-hour window (HFNC period) after HFNC initiation. Each HFNC trial was labeled either a failure (if there was an escalation within the associated HFNC period) or a success (if there was no such escalation within the associated HFNC period).

When a patient was discharged from the PICU within 24 hours of HFNC initiation, the target label was determined by the patient’s disposition at discharge (Textbox 2). Episodes with the dispositions *operating room*, *another hospital’s ICU*, or *another ICU in current hospital* were excluded as the outcome was ambiguous. HFNC trials associated with a favorable disposition (*general care floor*, *home*, or *step-down unit*) during the HFNC period were labeled as success.

**Target outcome mappings for high flow nasal cannula periods cut short by patient discharge.**

**Target outcome mapping and episode disposition**

**Success**

- General care floor
- Home
- Step-down unit or intermediate care unit

**Censored**

- Operating room
- Another hospital’s intensive care unit
- Another intensive care unit in current hospital

The HFNC definitions and outcomes, combined with the exclusion criteria, resulted in 834 HFNC trials that were randomly divided into training, validation, and hold-out test sets. All HFNC trials of an individual patient were assigned in only one of these 3 sets to prevent leakage and bias during model evaluations. No additional stratifications were applied.

### Labeling Time Series Data for Model Training and Assessment

**Figure 2:**
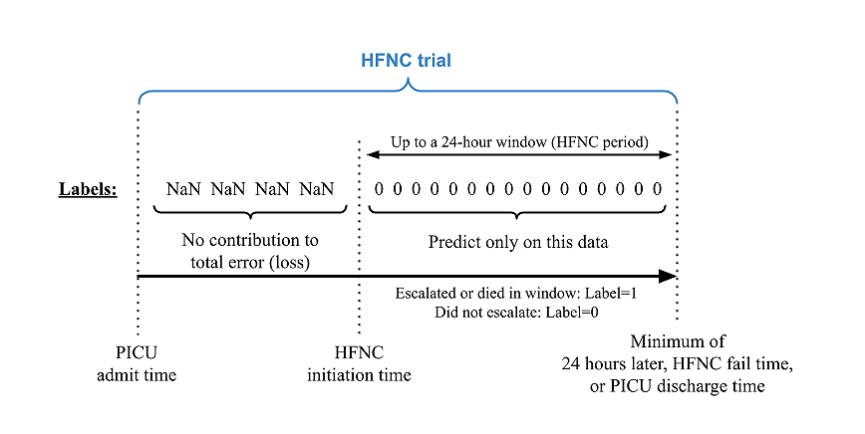
Illustration of labeling time series data for predicting HFNC escalation. HFNC: high nasal flow cannula; NaN: Not a Number; PICU: pediatric intensive care unit.

Recall that the task is to predict HFNC failure (escalation of care) for each HFNC trial. Data processing starts at the beginning of the HFNC trial, with a model trained to output a prediction each time a new measurement becomes available. Figure 2 illustrates how the time series data of each HFNC trial were labeled for this process. All prediction times during the HFNC period were labeled as either 1 (failure) or 0 (success). Predictions at times before the HFNC period were labeled *NaN* (*Not a Number*) to exclude the predictions from error metrics during model training and performance evaluations.

### Data Preprocessing

#### Overview

Each episode’s time series data were converted into a matrix. Rows contain the measurements (recorded or imputed) of all variables at 1 time point, and columns contain values of a single variable at different times. The steps of this conversion are described in detail in a previous work [15] and comprise the aggregation and normalization of observed measurements, followed by the imputation of missing data. A brief description is provided in the following sections.

#### Aggregation and Normalization

Where medically appropriate, values of the same variable obtained using independent measurement methods were aggregated into a single feature. For example, invasive and noninvasive systolic blood pressure measurements were grouped into a single variable representing the systolic blood pressure [16]. Any drug or intervention administered in <1% of patient episodes in the training set was excluded. This aggregation and exclusion process resulted in a list of 516 distinct demographic, physiological, laboratory, and therapy variables available as model inputs (see Tables S1 to S4 in Multimedia Appendices 1-4 for the full list; variable acronyms appear in Table S5 in Multimedia Appendix 5). Measurements considered incompatible with human life were filtered out using established minimum and maximum acceptable values (eg, heart rates >400 beats per minute). Physiological variables and laboratory measurements were transformed to have 0 mean and unit variance using the means and SDs derived from the training set. Administered patient therapies were scaled to the interval [0,1] using clinically defined upper limits. No variables were normalized by age as patient age was one of the inputs. Diagnoses were only used for descriptive analyses and not as model input features.

#### Imputation

EMR measurements were sparsely, asynchronously, and irregularly charted, with time between measurements ranging from 1 minute to several hours. At any time where at least one variable had a recorded value, the missing values for other unrecorded variables were imputed. The imputation process depended on the type of variable. Missing measurements for a drug or an intervention variable were set to 0, indicating the absence of treatment. When physiological observations or laboratory measurements were available, they were propagated forward until another measurement was recorded. This choice reflects the clinical practice and is based on the observation that measurements are recorded more frequently when the patient is unstable and less frequently when the patient appears stable [17]. If a physiological or laboratory variable had no recorded value throughout the entire episode, the mean from the training set population was used.

### Input Perseveration

As described in the study by Ledbetter et al [18], LSTMs exhibit predictive lag, wherein the model fails to react quickly to new clinical information. A previous study [18] demonstrated that an LSTM trained with input data perseveration (ie, the input is replicated *k* times) responds with more pronounced changes in predictions when new measurements become available while maintaining overall performance relative to a standard LSTM. As timely model responsiveness to acute clinical events is critical in determining the necessity of escalating support, input data perseveration was assessed as a training augmentation technique.

### Transfer Learning

TL is a technique of applying insights (eg, data representations) that were previously learned from one problem to a new, related task [19-22]. It can be particularly beneficial when one task has significantly more training data than the other. As the number of children on HFNC is significantly smaller than the number of ICU episodes in the CHLA PICU data set, TL techniques were considered to generate initial data representations and facilitate training of the HFNC prediction models. LSTM-based recurrent neural networks using the same input variables as those for the HFNC task were trained on >9000 CHLA PICU episodes to predict ICU mortality [23]. The first layer of one of these mortality models was then used as the first layer of some of the LSTM-based HFNC prediction models in Textbox 3.

**Details of the 8 models considered.**

**Model and hyperparameters (a list of the 14 variables used as model inputs appears in Table S6 of Multimedia Appendix 6)**

**14-variable logistic regression (LR-14)**

- Regularizer: 7.50×10^−1^
- Regularization: elasticnet (ratio=0.5)

**517-variable logistic regression (LR-517)**

- Regularizer: 1.15×10^−3^
- Regularization: elasticnet (ratio=0.2)

**Long short-term memory (LSTM)**

- Layers: 3
- Number of hidden units: (128,256,128)
- Batch size: 12
- Initial learning rate: 9.6e-4
- Patience: 10
- Reduce rate: 0.9
- Number of rate reductions: 8
- Loss function: binary cross-entropy
- Optimizer: rmsprop
- Dropout: 0.35
- Recurrent dropout: 0.2
- Regularizer: 1e-4
- Output activation: sigmoid

**LSTM with 3-times input perseveration (LSTM+3xPers**)

- Same as LSTM

**LSTM with transfer learning (TL; LSTM+TL)**

- Same as LSTM
- Transfer weights: first hidden layer only

**LSTM with 3-times input perseveration and TL (LSTM+3xPers+TL)**

- Same as LSTM
- Transfer weights: first hidden layer only

**Simple ensemble of LSTM+3xPers+TL (Simple-en-LSTM+3xPers+TL)**

- Same as LSTM
- Transfer weights: first hidden layer only

**Multi-ensemble of LSTM+3xPers+TL (Multi-EN-LSTM+3xPers+TL)**

- Same as LSTM
- Transfer weights: first hidden layer only

### Ensembling

Ensemble methods combine multiple algorithms to achieve a higher predictive performance than each component could obtain [24]. The predictions from each component are averaged to yield a single final prediction. Here, different seed values were used to generate multiple LSTM-based models, with each seed value initializing a different set of pseudorandom starting weights for a particular model. Different seed values led to slightly different models. Different seeds were used to train the mortality models used for TL and train the final LSTM models on HFNC-specific data. Owing to the relatively small size of the cohort available to develop the HFNC prediction model, it was hypothesized that training models with different seeds would result in high variance and low bias models that may be decorrelated. Ensembling provides a method to average the results across decorrelated models to reduce variance but maintain a low bias.

### HFNC Models

A total of 8 models were developed: a 14-variable LR (LR-14) using variables previously identified as risk factors for HFNC failure [8,11-13], a 517-variable LR (LR-517), a standard LSTM, an LSTM with input perseveration (LSTM+3xPers, where *3* indicates the number of replications described in the study by Ledbetter et al [18]), an LSTM with TL (LSTM+TL), an LSTM with both input perseveration and TL (LSTM+3xPers+TL), a simple ensemble of LSTMs with input perseveration and TL (Simple-EN-LSTM+3xPers+TL), and an ensemble of ensembles of LSTMs with input perseveration and TL (Multi-EN-LSTM+3xPers+TL). All models were trained to generate a prediction every time new measurements became available within the HFNC period.

Textbox 3 describes the parameters of all the models. Each model was developed on the training set to maximize the average of the validation set area under the receiver operating characteristic (AUROC) curves measured hourly from 0 to 14 hours into the HFNC period. This window was selected to prioritize the most clinically impactful period.

**Figure 3:**
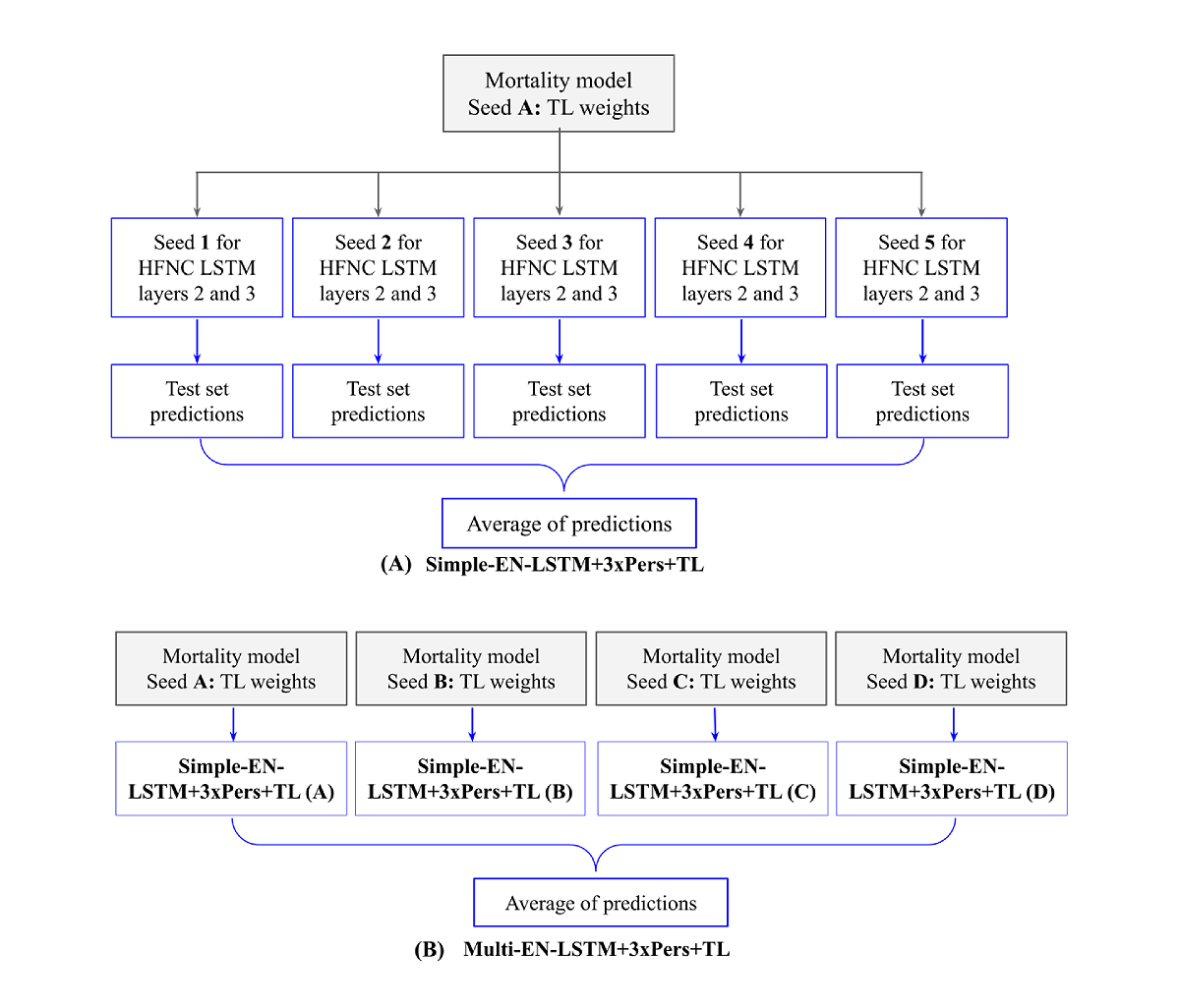
Forming the (A) simple ensemble and (B) multi-ensemble models. HFNC: high flow nasal cannula; LSTM: long short-term memory; TL: transfer learning.

Figure 3 illustrates how the ensemble models were formed. An LSTM mortality model was trained (*seed A*), and its first layer was used as the first layer (TL weights) of a 3-layer LSTM with input perseveration. Layers 2 and 3 of this model were trained on the HFNC data 5 times (*seeds 1-5*), resulting in 5 slightly different HFNC models whose predictions were averaged to generate the Simple-EN-LSTM+3xPers+TL model predictions. This process was repeated 4 times to generate an ensemble of ensembles model: 4 LSTM mortality models were trained (*seeds A-D*), each providing a different set of TL weights. For each of these 4 sets of TL weights, 5 different seeds were used to train with the HFNC data, resulting in 20 models whose predictions were averaged together to generate the Multi-EN-LSTM+3xPers+TL model predictions.

### Model Performance Assessment

Model performance was assessed on the test set by evaluating the AUROC of predictions every 30 minutes within the 24-hour HFNC period. AUROC performance for the subset of patients with respiratory diagnoses was also compared every 30 minutes within the 24-hour HFNC period. In the *rolling cohort* AUROC computations, failures or successes that had already occurred before the time of evaluation were excluded. For example, any HFNC failures or successes that took place ≤4.5 hours into the HFNC period were not considered in computing the 5-hour AUROC. Including these in the 5-hour AUROC calculation would artificially boost the result. This was followed for all time points of interest. Therefore, the number of HFNC failures and successes in the test set steadily decreased from 0 to 24 hours in the HFNC period. *The fixed cohort* AUROC and area under the precision–recall curve in the first 15 hours were also computed, wherein only those who were on HFNC for at least 15 hours were included to ensure a constant cohort (and, consequently, a constant incidence rate of HFNC failures) at each evaluation point.

In addition, receiver operating characteristic (ROC) curves, sensitivities, specificities, positive predictive values (PPVs), and negative predictive values (NPVs) of predictions 2 hours after HFNC initiation were generated to evaluate model performance early in the HFNC period, on both the entire test set cohort and the respiratory subcohort.

## Results

### Cohort Characteristics

**Figure 4:**
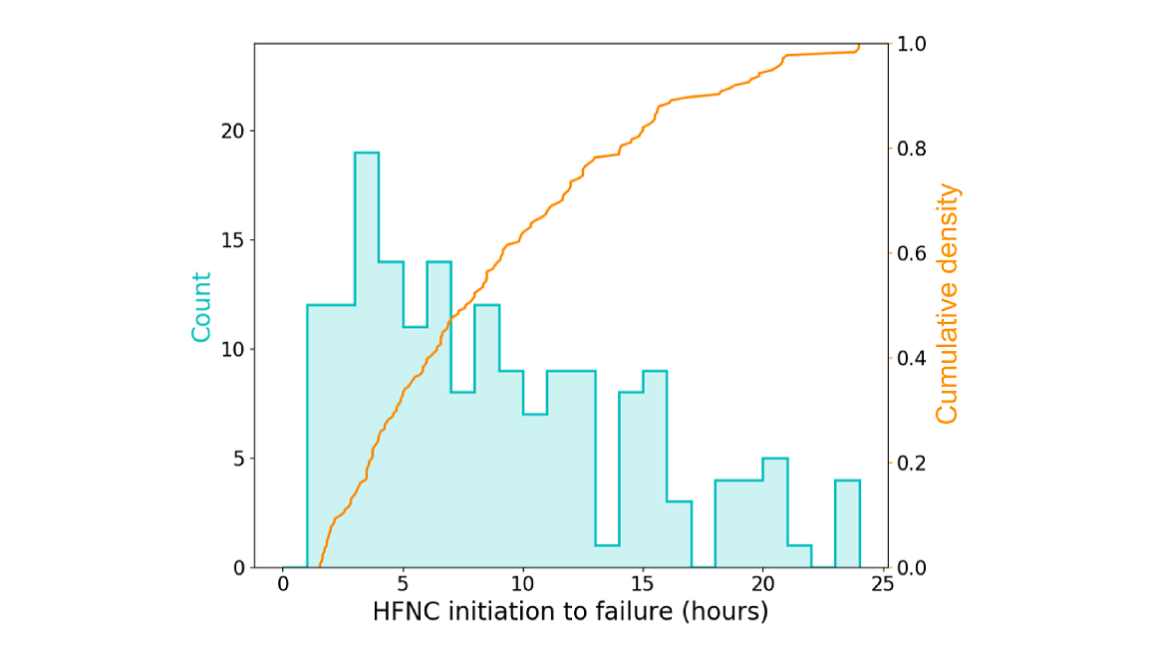
Distribution of time to HFNC failure. HFNC: high flow nasal cannula.

Table 1 describes the demographics and characteristics of the data, whereas Figure 4 shows the histogram (in cyan) and cumulative density (orange) for the time to HFNC failure for the entire data set. Approximately 50% (87/175) of failures occurred within 7.6 hours, and 80% (140/175) occurred within 14.1 hours.

**Table 1:** Demographics and characteristics of the data partitions (N=834).

Characteristic			Training set (n=455)		Validation set (n=173)		Test set (n=206)		Overall (n=834)
Patients, n			341		138		158		637
Episodes, n			381		151		183		715
HFNC^a^ trials died, n (%)			21 (4.6)		10 (5.8)		7 (3.4)		38 (4.6)
HFNC trials failed, n (%)			103 (22.6)		30 (17.3)		42 (20.4)		175 (21)
HFNC trials female, n (%)			200 (44)		70 (40.5)		90 (43.7)		360 (43.2)
HFNC trials with respiratory primary diagnosis, n (%)			333 (73.2)		115 (66.5)		141 (68.4)		589 (70.6)
**PRISM^b^ 3 score**									
	Values, mean (SD)	4.4 (5.3)		3.6 (5.0)		4.0 (5.0)		4.2 (5.2)	
	Values, median (IQR)	3 (0-7)		2 (0-5)		2 (0-6)		3 (0-6)	
**Age (years)**									
	Values, mean (SD)	3.3 (4.6)		3.1 (4.5)		2.8 (3.7)		3.1 (4.4)	
	Values, median (IQR)	1.2 (0.4-3.4)		1.2 (0.5-3.1)		1.2 (0.5-3.8)		1.2 (0.4-3.5)	
**Age group (years), n (%)**									
	0-1	205 (45.1)		78 (45.1)		96 (46.6)		379 (45.4)	
	1-5	164 (36)		63 (36.4)		77 (37.4)		304 (36.5)	
	5-10	31 (6.8)		12 (6.9)		17 (8.3)		60 (7.2)	
	10-19	55 (12.1)		20 (11.6)		16 (7.8)		91 (10.9)	

^a^HFNC: high flow nasal cannula.

^b^PRISM: pediatric risk of mortality.

### AUROC Across the First 24 Hours

**Figure 5:**
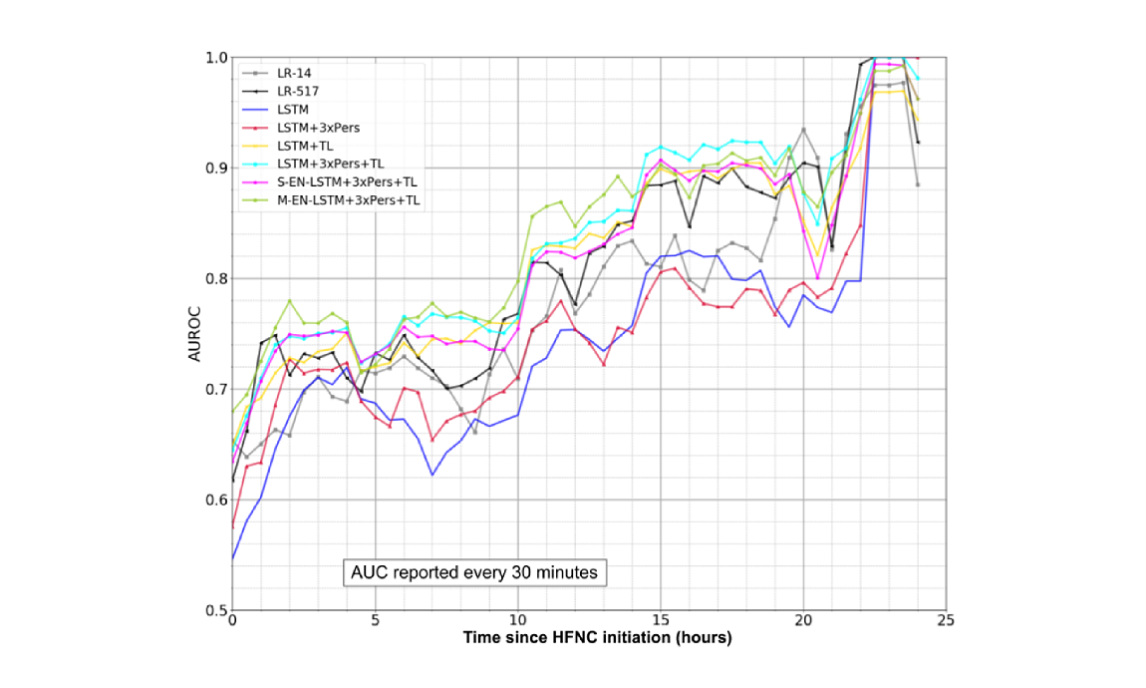
Area under the receiver operating characteristics (AUROCs) of model predictions at different times on hold-out test set. AUC: area under the receiver operating characteristic curve; HFNC: high flow nasal cannula; LR: logistic regression; LSTM: long short-term memory; TL: transfer learning.

Figure 5 shows the 8 models’ *rolling cohort* AUROCs in 30-minute increments within the 24-hour HFNC period of all HFNC trials in the test set. Table S7 in Multimedia Appendix 7 shows the number of remaining HFNC trials in the test cohort at various evaluation times. Table S8 in Multimedia Appendix 8 presents the test set AUROC values associated with Figure 5 at several times of interest in the first 12 hours of the HFNC period. Table S9 in Multimedia Appendix 9 presents the corresponding AUROCs in the respiratory cohort. The *fixed cohort* AUROCs and areas under the precision–recall curve are shown in Figures S10 and S11 in Multimedia Appendices 10 and 11. Both the rolling and fixed cohort AUROCs generally increased over time.

### Two-Hour ROC and AUROC

**Figure 6:**
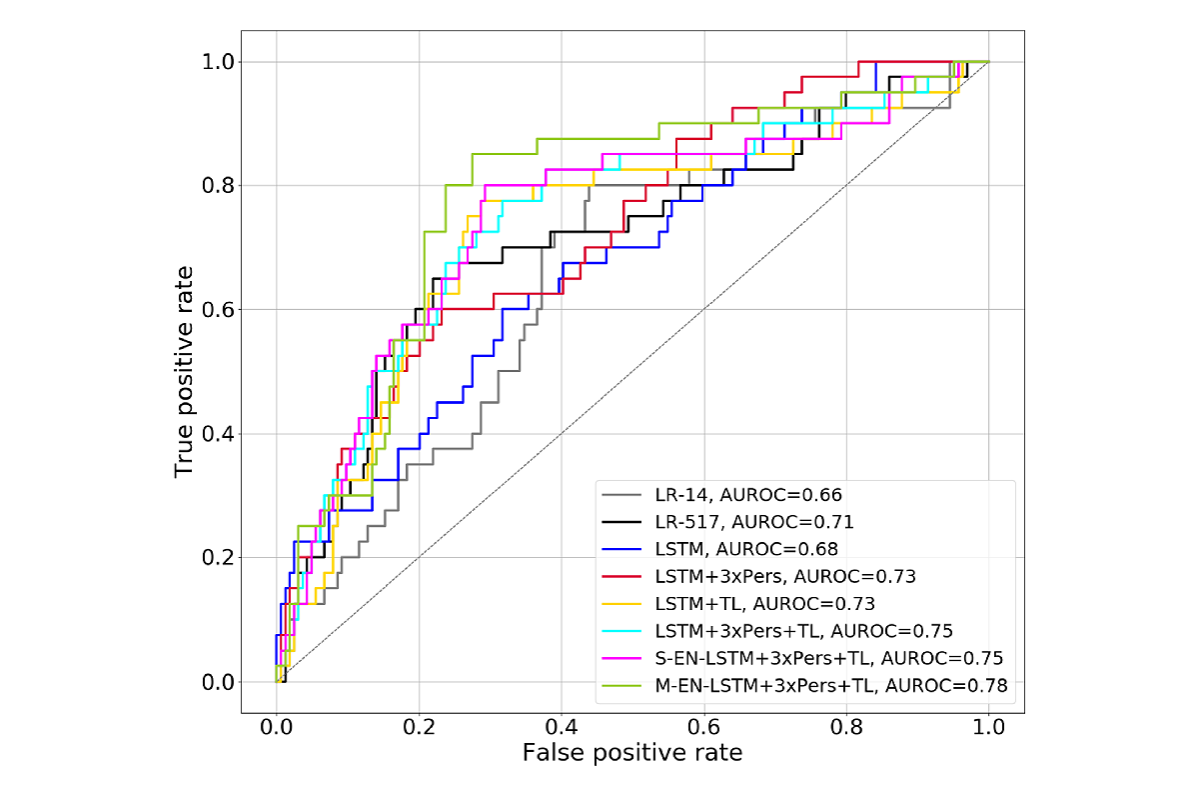
Receiver operating characteristic curves and area under the receiver operating characteristic (AUROC) curves of 2-hour predictions on the entire test set. LR: logistic regression; LSTM: long short-term memory; TL: transfer learning.

**Figure 7:**
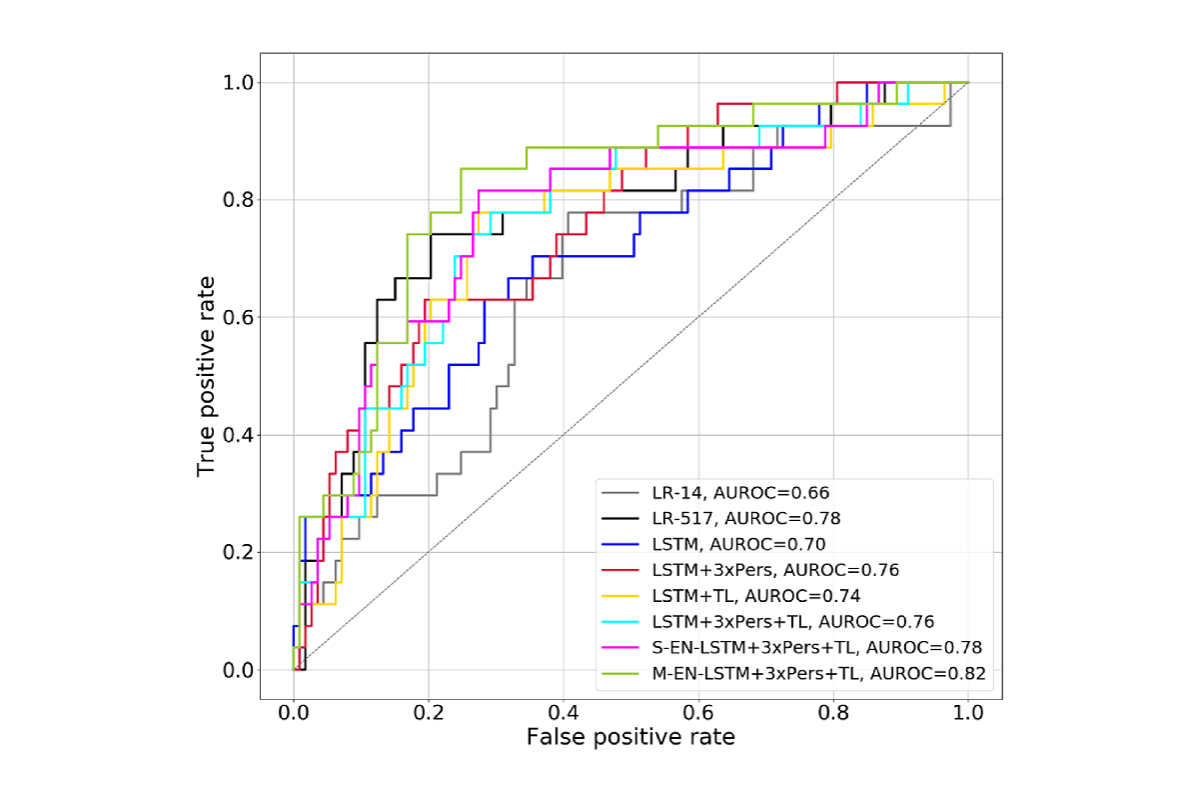
Receiver operating characteristic curves and area under the receiver operating characteristic (AUROC) curves of 2-hour predictions on high flow nasal cannula trials whose primary diagnosis is respiratory: all models. LR: logistic regression; LSTM: long short-term memory; TL: transfer learning.

Figure 6 presents the test set 2-hour ROC curves and AUROC for the 8 models, showing predictive performance just 2 hours into the HFNC period, whereas Figure 7 presents the same metrics corresponding to the respiratory cohort.

### Positive Predictive Value and Negative Predictive Value

Table 2 shows the specificity, PPV, and NPV for the 2-hour predictions of the Multi-EN-LSTM+3xPers+TL model that correspond to different values of sensitivity. These metrics provide a more intuitive understanding of performance in a deployment scenario. At the 2-hour mark, 204 HFNC periods remained (40 [19.6%] failures and 164 [80.4%] successes); setting the operating point at 25% sensitivity correctly identified 10 of the HFNC failures (PPV=67%) and 159 of the nonfailures (NPV=84%). Among the correctly identified HFNC failures, the time to failure (at the 2-hour mark) ranged from a few minutes to 19 hours (median=2.6 hours). Tables S12 to S14 in Multimedia Appendices 12-14 show a comparison of these metrics across all models.

**Table 2:** Specificity, PPV,^a^ and NPV^b^ corresponding to various sensitivity values of the 2-hour predictions of the Multi-EN-LSTM+3xPers+TL model.

Sensitivity	Specificity	PPV	NPV
0.10	0.982	0.571	0.817
0.20	0.970	0.615	0.832
0.25	0.970	0.667	0.841
0.30	0.927	0.500	0.844
0.40	0.848	0.390	0.853
0.50	0.835	0.426	0.873
0.60	0.793	0.414	0.890
0.70	0.793	0.452	0.915
0.80	0.762	0.451	0.940
0.90	0.463	0.290	0.950
0.95	0.207	0.226	0.944
1.00	0.049	0.204	1.000

^a^PPV: positive predictive value.

^b^NPV: negative predictive value.

## Discussion

### Principal Findings

The ability to predict a child’s response to HFNC reliably and in real time could help guide clinical differentiation among three groups: (1) children most likely to do well on HFNC alone, (2) children most likely to need a higher level of support, and (3) children whose likely HFNC outcome is unclear and who may require additional observation. Patients identified from the first group may require less clinical intervention and free up scarce ICU resources. Identifying children in the second group may enable clinicians to intervene more rapidly and provide adequate support to prevent decompensation. Owing to clinical uncertainty, children in the third group may benefit from more careful and frequent observation with the continuous prediction of the likelihood of failure.

The granular longitudinal data captured from children in the ICU presents a tremendous amount of information available for learning and developing tools to help differentiate children’s responses to numerous ICU interventions such as HFNC, including ventilation, extracorporeal membrane oxygenation, and dialysis. Deep learning models, especially those with sequential processing capabilities such as LSTMs, have the potential to use rich time-dependent data in ways that more traditional machine learning models (eg, LR) cannot; however, LSTMs may require sizable training data to construct generalizable models. The results from this study showed this to be the case: a standard, 3-layer LSTM was generally the worst performing model on the hold-out test set.

TL was incorporated to address the issue of training LSTMs with insufficient data. The models with TL had the advantage of learning representations from >9000 PICU episodes, whereas the models without TL learned from >600 HFNC trials (approximately 500 episodes). The results demonstrate considerable gains from using TL and are consistent with the theory [14]. Figure 5, Figure S10, and Table S8, in particular, highlight the significant and time-independent performance increase delivered by TL in the LSTM models.

Input perseveration by itself (LSTM+3xPers) provided a performance boost relative to the standard LSTM, especially in the first 12 hours of the HFNC period in respiratory patients (Table S9, Multimedia Appendix 9). When combined with TL (LSTM+3xPers+TL), it continued to provide additional, although slight, gains. As demonstrated in the study by Ledbetter et al [18], LSTMs can exhibit a predictive lag phenomenon, wherein they fail to react rapidly to new data reflecting sudden clinical events and changes in patient status. In the context of HFNC use and decisions about whether to escalate a child to higher levels of support, this predictive lag may be deleterious in a time-constrained environment such as the PICU.

Finally, the ensemble models (Simple-EN-LSTM+3xPers+TL and Multi-EN-LSTM+3xPers+TL) were built to address another consequence of limited training data: the relatively high variability of any one particular realization of the model. This is a byproduct of randomly chosen initialization seeds used to initialize LSTM weights and biases and for random dropout techniques used for regularization purposes. The ensemble methods provided a consistently higher performance on the hold-out test set than the nonensemble models. The ensemble models provided a slight performance boost over just a single LSTM+3xPers+TL model. Not surprisingly, the multiensembling of multiple models (both of those used to generate TL weights and those used to generate HFNC predictions) provided the best overall model (Multi-EN-LSTM+3xPers+TL).

Regardless of the model, the performance generally increased over time (Figure 5 and Figure S10). This is not surprising as the *lead time* (the interval between the times of prediction and outcome) decreases [25].

Model performance in patients with respiratory diagnoses is of interest as the pathophysiology of respiratory illness is particularly amenable to HFNC therapy [1-4]. Approximately 70% of HFNC initiation in this cohort were in patients with respiratory illnesses. Table S8 shows that all models except LR-14 generally performed better in the respiratory group over time. Figure 7 shows that the best performing models in the overall cohort—those that incorporated TL—performed even better in the respiratory group after 2 hours of observation, demonstrating the TL models’ potential clinical impact.

The 2-hour mark after HFNC initiation is an important clinical decision point as a child has had adequate time to adapt to HFNC, and the effects of treatment can be assessed. This motivated the additional analyses of 2-hour predictions shown in Figure 6 and Figure 7 (ROC curves) and Table 2 (sensitivity, specificity, PPV, and NPV at various decision thresholds). The Multi-EN-LSTM+3xPers+TL model had the highest AUROC. In this model’s ROC curve for the entire cohort, 2 operating points are of particular interest: the first corresponds to 95% sensitivity (20% specificity), and the second corresponds to 25% sensitivity (97% specificity, 67% PPV, and 84% NPV). The first point can be used to identify children most likely to do well in HFNC (group 1), whereas the second can identify those most likely to fail HFNC (group 2). Successfully identifying 20% of group 1 can reduce the observational burden, whereas identifying 25% of group 2 could lead clinicians to intervene earlier with an escalation to a higher level of O_2_ support, potentially improving outcomes for these children [9,10]. This system could potentially enable intervention 2 to 3 hours earlier in those most likely to fail HFNC. Children for whom the model predictions fall between the 2 thresholds are in the third group: those whose HFNC outcome is unclear and who may benefit from more frequent observations.

### Limitations

This study had several limitations. First, it was based on a single-center retrospective cohort. Second, the target definition considered only the first 24 hours following HFNC initiation. Further work can refine the target to consider the subsequent 24 hours, regardless of how long the patient has already been on HFNC.

Finally, this study is limited by the exclusion of children experiencing apnea, making the predictive model’s applicability to such children unclear. Although less than ideal, this exclusion was deemed necessary as it is difficult to determine whether escalation to BiPAP in these children is because of clinical necessity (ie, true escalation) or prophylactic caution (to guard against sleep apnea).

### Conclusions

This study demonstrated the feasibility of applying advanced machine learning methodology to a complex and challenging clinical situation. This work demonstrated that clinically relevant models can be trained to predict the risk of escalation from HFNC within 24 hours of initiation of therapy and could be obtained by using an LSTM with the application of TL and input perseveration to boost AUROC performance.

## Appendix

Multimedia Appendix 1 Demographic variables and vital observations used as input variables for long short-term memory models. See Table S5 for acronym expansions.

Multimedia Appendix 2 Laboratory and echocardiogram measurements used as input variables for long short-term memory models. See Table S5 for acronym expansions.

Multimedia Appendix 3 Drugs used as input variables for long short-term memory models. See Table S5 for acronym expansions.

Multimedia Appendix 4 Interventions used as input variables for long short-term memory models. See Table S5 for acronym expansions.

Multimedia Appendix 5 Acronyms and abbreviations used in Tables S1 to S4.

Multimedia Appendix 6 List of the input variables used in the 14-variable logistic regression model.

Multimedia Appendix 7 Number of high flow nasal cannula trials remaining at various evaluation points.

Multimedia Appendix 8 Test set area under the receiver operating characteristics (AUROCs) curve of the 8 models considered at various time points following high flow nasal cannula initiation. The highest AUROC in a column is in bold font.

Multimedia Appendix 9 Test set area under the receiver operating characteristics (AUROCs) curve of the 8 models considered at various time points following high flow nasal cannula initiation for children with a respiratory diagnosis. The highest AUROC in a column is in bold font.

Multimedia Appendix 10 Area under the receiver operating characteristics (AUROCs) curve of model predictions over the first 15 hours for a fixed set of patients with at least 15 hours of data on high flow nasal cannula on the holdout test set.

Multimedia Appendix 11 Area under the precision recall curves (AUPRCs) of model predictions over the first 15 hours for a fixed set of patients with at least 15 hours of data on high flow nasal cannula on the holdout test set.

Multimedia Appendix 12 Sensitivity versus specificity of the 2-hour predictions in the entire test set. The highest specificity along each row (fixed sensitivity) is in bold.

Multimedia Appendix 13 Sensitivity versus positive predictive value of the 2-hour predictions in the entire test set. The highest positive predictive value along each row (fixed sensitivity) is in bold.

Multimedia Appendix 14 Sensitivity versus negative predictive value of the 2-hour predictions in the entire test.

## Abbreviations

AUROC: area under the receiver operating characteristic
BiPAP: bilevel positive airway pressure
CHLA: Children’s Hospital Los Angeles
EMR: electronic medical record
HFNC: high flow nasal cannula
ICU: intensive care unit
LR: logistic regression
LR-14: 14-variable logistic regression
LR-517: 517-variable logistic regression
LSTM: long short-term memory
ML: machine learning
NIV: noninvasive ventilation
NPV: negative predictive value
PICU: pediatric intensive care unit
PPV: positive predictive value
ROC: receiver operating characteristic
TL: transfer learning
