# Supplementary material for: Predicting High Flow Nasal Cannula Failure in an Intensive Care Unit Using a Recurrent Neural Network With Transfer Learning and Input Data Perseveration: Retrospective Analysis
Source: JMIR Med Inform. 2022 Mar 3;10(3):e31760. doi: 10.2196/31760 (PMC8931642; doi:10.2196/31760)
Supplement: Multimedia Appendix 1 [file medinform_v10i3e31760_app1.docx]

**Table A-1.** Demographic variables and vital observations used as input variables for LSTM models. See Table A-5 for acronym expansions.

| **Demographics, Vitals & Misc. Measurements** | | |
| --- | --- | --- |
| sf_ratio | Abdominal Assessment_soft | Abdominal Assessment_firm |
| Abdominal Assessment_tender | Abdominal Assessment_distended | Abdominal Assessment_round |
| Abdominal Assessment_flat | Abdominal Assessment_edematous | Abdominal Girth |
| Activity Level | Behavioral Assessment_sleeping | Behavioral Assessment_awake_quiet |
| Behavioral Assessment_disrupted_sleep | Behavioral Assessment_sedated | Behavioral Assessment_occasional_cry |
| Behavioral Assessment_active | Behavioral Assessment_no_response | Behavioral Assessment_restless |
| Behavioral Assessment_agitation | Behavioral Assessment_drowsy | Behavioral Assessment_posturing |
| Behavioral Assessment_grimaces | Behavioral Assessment_verbal | Bladder pressure |
| Bowel Sounds | Breath Sounds_coarse | Breath Sounds_coarse_crackles |
| Breath Sounds_fine_crackles | Breath Sounds_moist crackles | Breath Sounds_scattered |
| Breath Sounds_rhonchi | Breath Sounds_wheezing | Breath Sounds_diminished |
| Breath Sounds_diffuse | Breath Sounds_squeaks | Breath Sounds_clear |
| Capillary Refill Rate | Capillary Refill Delayed | Central Venous Pressure |
| Cerebral Perfusion Pressure | Cough Present | Diastolic Blood Pressure |
| EtCO2 | ETT Airway Leak | Extremity Temperature Level |
| Eye Response Level | FLACC Pain Activity | FLACC Pain Consolability |
| FLACC Pain Cry | FLACC Pain Face | FLACC Pain Legs |
| FLACC Pain Intensity | Gag Present | Glasgow Coma Score |
| Head Circumference | Heart Rate | Heart Sounds_murmur |
| Heart Sounds_gallop | Heart Sounds_normal | Height |
| Intracranial Pressure | Left Pupil Size Before Light | Left Pupil Size After Light |
| Left Pupillary Response Level | Level of Consciousness | Lip Moisture Level |
| Mean Arterial Pressure | Motor Response Level | Nasal Flaring Level |
| Nutrition Level | Oxygenation Index | PaO2 to FiO2 |
| Patient Mood Level | Peripheral Intravenous Line Site | Plasma Hemoglobin |
| Potential for Pain_disease_process | Potential for Pain_invasive_line | Potential for Pain_postop |
| Potential for Pain_postop24hr | Potential for Pain_chest_tube | Potential for Pain_burn |
| Potential for Pain_ventriculostomy | Potential for Pain_fracture | Pulse Oximetry |
| Quality of Pain Level | Respiratory Effort Level | Respiratory Rate |
| Right Pupil Size Before Light | Right Pupil Size After Light | Right Pupillary Response Level |
| Sedation Scale Level | Side Rails | Skin Integrity_dry |
| Skin Turgor_edema | Skin Turgor_turgor | Systolic Blood Pressure |
| Temperature | Verbal Response Level | WAT1 Total |
| Weight | Heart Rate_ecg | printerval |
| qtinterval | qtcb | pfrontaxis |
| i40frontaxis | t40frontaxis | qrsfrontaxis |
| stfrontaxis | tfrontaxis | phorizaxis |
| i40horizaxis | t40horizaxis | qrshorizaxis |
| sthorizaxis | rrinterval | thorizaxis |
| pduration | qonset | tonset |
| qtcf | qrsduration | Age |
| race_Black or African American | race_Hispanic or Latino | race_White |
| race_Asian/Indian/Pacific Islander | race_unknown | Sex_M |
| Sex_F | deltat^*^ |  |

^*^deltat is the time step between two data recordings for a given episode or HFNC trial
