# Supplementary material for: Predicting High Flow Nasal Cannula Failure in an Intensive Care Unit Using a Recurrent Neural Network With Transfer Learning and Input Data Perseveration: Retrospective Analysis
Source: JMIR Med Inform. 2022 Mar 3;10(3):e31760. doi: 10.2196/31760 (PMC8931642; doi:10.2196/31760)
Supplement: Multimedia Appendix 2 [file medinform_v10i3e31760_app2.docx]

**Table A-2.** Laboratory and echocardiogram measurements used as input variables for LSTM models. See Table A-5 for acronym expansions.

| **Labs and Echocardiogram** | | |
| --- | --- | --- |
| ABG Base excess | ABG FiO2 | ABG HCO3 |
| ABG O2 sat | ABG PCO2 | ABG PO2 |
| ABG TCO2 | ABG pH | ALT |
| AST | Albumin Level | Alkaline phosphatase |
| Amylase | Antithrombin III Function | Anti-Xa Heparin |
| B-type Natriuretic Peptide | BUN | Bands % |
| Basophils % | Bicarbonate Serum | Bilirubin Conjugated |
| Bilirubin Total | Bilirubin Unconjugated | Blasts % |
| C-Reactive Protein | Cardiac Rhythm_normal_sinus | Cardiac Rhythm_sinus_tachycardia |
| Cardiac Rhythm_sinus_bradycardia | Cardiac Rhythm_bundle_branch_block | Cardiac Rhythm_pac |
| Cardiac Rhythm_pvc | CBG Base excess | CBG FiO2 |
| CBG HCO3 | CBG O2 sat | CBG PCO2 |
| CBG PO2 | CBG TCO2 | CBG pH |
| CSF Color_xanthochromic | CSF Lymphs % | CSF RBC |
| CSF Segs % | CSF WBC | CSF Glucose |
| CSF Protein | Calcium Ionized | Calcium Total |
| Chloride | Complement C3 Serum | Complement C4 Serum |
| Creatinine | Culture CSF | Culture Blood |
| Culture Fungus Blood | Culture Respiratory | Culture Urine |
| Culture Wound | ESR | Eosinophils % |
| Ferritin Level | Fibrinogen | GGT |
| Glucose | Haptoglobin | Hematocrit |
| Hemoglobin | INR | Influenza Lab |
| Lactate | Lactate Dehydrogenase Blood | Lactic Acid Blood |
| Lipase | Lymphocyte % | MCH |
| MCHC | MCV | MVBG Base Excess |
| MVBG HCO3 | MVBG O2 Sat | MVBG PCO2 |
| MVBG PO2 | MVBG TCO2 | MVBG pH |
| Macrocytes | Magnesium Level | Metamyelocytes % |
| Monocytes % | Myelocytes % | Neutrophils % |
| PT | PTT | Phosphorus level |
| Platelet Count | Potassium | Protein Total |
| RBC Blood | RDW | Reticulocyte Count |
| Schistocytes | Sodium | Spherocytes |
| T4 Free | TSH | Triglycerides |
| VBG Base excess | VBG FiO2 | VBG HCO3 |
| VBG O2 sat | VBG PCO2 | VBG PO2 |
| VBG TCO2 | VBG pH | White Blood Cell Count |
| Ao max PG | TR max PG | TR max vel |
| PV V2 max | Med Peak A' Vel | LVPWd(MM) |
| IVSd(MM) | MV dec time | Med Peak E' Vel |
| MV E/A | FS(MM) | MV A max vel |
| LVIDd(MM) | Med Peak S Vel | E/E' med |
| MV E max vel | LVIDs(MM) | MV Lat Peak S' Vel |
| MV Lat Peak E' Vel | E/E' lat (MV) | PI max vel |
| PI max PG | ESV(MOD-sp4) | Tei Index LV (LV MPI) |
| TV A max vel | TV E/A | TV E max vel |
| RV-0056 | RV-0001 | LV-0001 |
| LV-0198 | RV-0065 | RV-0021 |
| RV-0016 | LV-0027 | LV-0026 |
| LV-0029 |  |  |
