# Supplementary material for: Predicting High Flow Nasal Cannula Failure in an Intensive Care Unit Using a Recurrent Neural Network With Transfer Learning and Input Data Perseveration: Retrospective Analysis
Source: JMIR Med Inform. 2022 Mar 3;10(3):e31760. doi: 10.2196/31760 (PMC8931642; doi:10.2196/31760)
Supplement: Multimedia Appendix 3 [file medinform_v10i3e31760_app3.docx]

**Table A-3.** Drugs used as input variables for LSTM models. See Table A-5 for acronym expansions.

| **Drugs** | | |
| --- | --- | --- |
| inotrope_score | Acetaminophen_inter | Acetazolamide_inter |
| Acyclovir_inter | Albumin_inter | Albuterol_inter |
| Alteplase_inter | Amikacin_inter | Aminophylline_cont |
| Aminophylline_inter | Amlodipine_inter | Amoxicillin/clavulanic acid_inter |
| Amoxicillin_inter | Amphotericin B Lipid Complex_inter | Ampicillin/Sulbactam_inter |
| Ampicillin_inter | Aspirin_inter | Atropine_inter |
| Azithromycin_inter | Baclofen_inter | Budesonide_inter |
| Bumetanide_inter | Calcium Chloride_cont | Calcium Chloride_inter |
| Calcium Gluconate_inter | Cefazolin_inter | Cefepime_inter |
| Cefotaxime_inter | Ceftazidime_inter | Ceftriaxone_inter |
| Cephalexin_inter | Chlorothiazide_inter | Ciprofloxacin HCL_inter |
| Cisatracurium_cont | Cisatracurium_inter | Clindamycin_inter |
| Clonidine HCl_inter | Cyclophosphamide_inter | Desmopressin_inter |
| Dexamethasone_inter | Dexmedetomidine_cont | Diazepam_inter |
| Digoxin_inter | Diphenhydramine HCl_inter | Dopamine_cont |
| Enalapril_inter | Enoxaparin_inter | Epinephrine_cont |
| Epinephrine_inter | Epoetin_inter | Famotidine_inter |
| Fentanyl_cont | Fentanyl_inter | Ferrous Sulfate_inter |
| Filgrastim_inter | Fluconazole_inter | Fluticasone_inter |
| Furosemide_cont | Furosemide_inter | Gabapentin_inter |
| Ganciclovir Sodium_inter | Gentamicin_inter | Glycopyrrolate_inter |
| Heparin_cont | Heparin_flush_inter | Heparin_inter |
| Hydrocortisone_inter | Hydromorphone_cont | Hydromorphone_inter |
| Ibuprofen_inter | Immune Globulin_inter | Insulin_cont |
| Insulin_inter | Ipratropium Bromide_inter | Isradipine_inter |
| Ketamine_cont | Ketamine_inter | Ketorolac_inter |
| Labetalol_inter | Lactobacillus_inter | Lansoprazole_inter |
| Levalbuterol_inter | Levetiracetam_inter | Levocarnitine_inter |
| Levofloxacin_inter | Levothyroxine Sodium_inter | Lidocaine_inter |
| Lorazepam_inter | Magnesium Sulfate_inter | Mannitol_inter |
| Meropenem_inter | Methadone_inter | Methylprednisolone_inter |
| Metoclopramide_inter | Metronidazole_inter | Micafungin_inter |
| Midazolam HCl_cont | Midazolam HCl_inter | Milrinone_cont |
| Morphine_inter | Mycophenolate Mofetl_inter | Naloxone HCL_inter |
| Nifedipine_inter | Norepinephrine_cont | Nystatin_inter |
| Olanzapine_inter | Ondansetron_inter | Oseltamivir_inter |
| Oxacillin_inter | Oxcarbazepine_inter | Oxycodone_inter |
| Pantoprazole_inter | Penicillin G Sodium_inter | Pentobarbital_inter |
| Phenobarbital_inter | Piperacillin/Tazobactam_inter | Potassium Chloride_inter |
| Potassium Phosphate_inter | Prednisolone_inter | Prednisone_inter |
| Propofol_cont | Propofol_inter | Propranolol HCl_inter |
| Quetiapine_inter | Racemic Epi_inter | Ranitidine_inter |
| Risperidone_inter | Rocuronium_inter | Sildenafil_inter |
| Sodium Bicarbonate_inter | Sodium Chloride_inter | Sodium Phosphate_inter |
| Spironolactone_inter | Sucralfate_inter | Tacrolimus_inter |
| Terbutaline_cont | Tobramycin_inter | Topiramate_inter |
| Trimethoprim/Sulfamethoxazole_inter | Ursodiol_inter | Valproic Acid_inter |
| Vancomycin_inter | Vecuronium_inter | Vitamin K_inter |
| Voriconazole_inter |  |  |
