# Supplementary material for: Predicting High Flow Nasal Cannula Failure in an Intensive Care Unit Using a Recurrent Neural Network With Transfer Learning and Input Data Perseveration: Retrospective Analysis
Source: JMIR Med Inform. 2022 Mar 3;10(3):e31760. doi: 10.2196/31760 (PMC8931642; doi:10.2196/31760)
Supplement: Multimedia Appendix 4 [file medinform_v10i3e31760_app4.docx]

**Table A-4.** Interventions used as input variables for LSTM models. See Table A-5 for acronym expansions.

| **Interventions** | |
| --- | --- |
| Abdominal X Ray | HFOV Amplitude |
| Arterial Line Site | Central Venous Line Site |
| Chest Tube | Comfort Response Level |
| CT Abdomen Pelvis | CT Brain |
| CT Chest | Chest X Ray |
| Pharmacological Comfort Measures Given | Continuous EEG Present |
| Diversional Activity_music | Diversional Activity_tv |
| Diversional Activity_play | Diversional Activity_toys |
| Diversional Activity_books | EPAP |
| EVD Clamp | FiO2 |
| HFOV Frequency | Gastrostomy Tube Location |
| Hemofiltration Therapy Mode | IPAP |
| Inspiratory Time | Mechanical Ventilation Mode |
| MRI Brain | Mean Airway Pressure |
| MultiDisciplinaryTeam Present | NIV Mode |
| NIV Set Rate | Nitric Oxide |
| Nurse Activity Level Completed_bedrest | Nurse Activity Level Completed_repositioned |
| Nurse Activity Level Completed_logroll | Nurse Activity Level Completed_microturns |
| Nurse Activity Level Completed_swaddled | Nurse Activity Level Completed_turn |
| Nurse Activity Level Completed_TV | Nurse Activity Level Completed_held |
| Nurse Activity Level Completed_chair | Nurse Activity Level Completed_swing |
| Nurse Activity Level Completed_infantseat | Nurse Activity Level Completed_dangle |
| Nurse Activity Level Completed_commode | Nurse Activity Level Completed_wheelchair |
| Nurse Activity Level Completed_stroller | Nurse Activity Level Completed_ambulate |
| Nurse Activity Level Completed_outofbed | Nurse Activity Level Completed_play |
| O2 Flow Rate | O2Support_1_NasalCannula |
| O2Support_2_SimpleMask | O2Support_3_PartialRebreather |
| O2Support_4_NonRebreather | O2Support_5_HighFlowNasalCannula |
| O2Support_6_NonInvasiveVent | O2Support_7_TTubePiece |
| O2Support_8_Ventilator | O2Support_9_HFOV |
| Oxygen Therapy | Oxygen Mode Level |
| PEEP | Peak Inspiratory Pressure |
| Position Support Given | Position Tolerance Level |
| Pressure Support | Psychiatry Consult |
| Range of Motion Assistance Type | Sedation Intervention Level |
| Sedation Response Level | Tidal Volume Delivered |
| Tidal Volume Expiratory | Tidal Volume Inspiratory |
| Tidal Volume Set | Tracheostomy Tube Size |
| Ventilator Rate | Ventriculostomy Site |
| Visitor Mood Level | Visitor Present_mother |
| Visitor Present_father | Visitor Present_grandmother |
| Visitor Present_sibling | Visitor Present_aunt |
| Visitor Present_uncle | Visitor Present_grandfather |
| Visitor Present_friend | Visitor Present_stepfather |
| Visitor Present_stepmother | Volume Tidal |
