# Supplementary material for: Predicting High Flow Nasal Cannula Failure in an Intensive Care Unit Using a Recurrent Neural Network With Transfer Learning and Input Data Perseveration: Retrospective Analysis
Source: JMIR Med Inform. 2022 Mar 3;10(3):e31760. doi: 10.2196/31760 (PMC8931642; doi:10.2196/31760)
Supplement: Multimedia Appendix 5 [file medinform_v10i3e31760_app5.docx]

**Table A-5** Acronyms and Abbreviations Used in Tables A-1 – A-4.

| **Acronyms and Abbreviations** | |
| --- | --- |
| ABG | Arterial Blood Gas |
| ALT | Alanine Aminotransferase |
| AST | Aspartate Transminase |
| AUROC | Area Under the Receiver Operating Characteristic Curve |
| BUN | Blood Urea Nitrogen |
| CBG | Capillary Blood Gas |
| cont | Continuous drug infusion |
| CSF | Cerebral Spinal Fluid |
| CT | Computerized Tomography |
| E/E’ lat (MV) | MV E Point / Lat Peak E Vel |
| E/E’ med | MV A point / Med Peak E Vel |
| ECMO | Extracorporeal Membrane Oxygenation |
| EMR | Electronic Medical Record |
| EPAP | Expiratory Positive Airway Pressure |
| ESR | Erythrocyte Sedimentation Rate |
| ESV(MOD-sp4) | Trace the left ventricular volume contour at end-systole (apical 4-chamber view) |
| EVD | External ventricular drain |
| EtCO2 | End-tidal Carbon Dioxide |
| FiO2 | Fraction of Inspired Oxygen |
| FLACC | Face, Legs, Activity, Cry, Consolability (pain score) |
| GGT | Gamma Glutanyl Transferase |
| HCO3 | Bicarbonate |
| HFOV | High Frequency Oscillatory Ventilation |
| INR | International Normalized Ratio |
| inter | Intermittent drug infusion |
| IPAP | Inspiratory Positive Airway Pressure |
| IVSd (MM) | End-diastolic septal thickness (M-mode) |
| LSTM | Long Short Term Memory |
| LV - 0001 | Normal LV structure and size |
| LV - 0002 | Dilated left ventricle |
| LV - 0004 | concentric LVH |
| LV - 0026 | Normal left ventricular diastolic function |
| LV - 0027 | Hyperdynamic left ventricular function |
| LV - 0029 | Abnormal left ventricular diastolic function |
| LV - 0198 | Underfilled LV |
| LV - 0208 | Decreased LV systolic function |
| LVIDd (MM) | Left ventricular internal dimension at end-diastole (M-mode) |
| LVIDs (MM) | Left ventricle, End-systolic dimension (M-mode) |
| LVPWd | Left ventricular posterior wall at end-diastole |
| MCH | Mean Corpuscular Hemoglobin |
| MCHC | Mean Corpuscular Hemoglobin Concentration |
| MCV | Mean Corpuscular Volume |
| MRI | Magnetic Resonance Imaging |
| MVBG | Mixed Venous Blood Gas |
| MV A max vel | A-point maximum velocity of the mitral flow |
| MV E max vel | E-point maximum velocity of the mitral flow |
| MV E/A | (MV A max vel) / (MV A max vel) |
| MV dec time | Mitral deceleration time |
| MV Lat Peak A’ Vel | Peak A-wave velocity of the left ventricular lateral mitral valve annulus tissue doppler spectrum |
| MV Lat Peak E’ Vel | Peak E-wave velocity of the lateral mitral valve annulus tissue doppler spectrum |
| MV Lat Peak S’ Vel | Peak S-wave velocity of the lateral mitral valve annulus tissue doppler spectrum |
| MV max PG | Maximum pressure gradient of the flow distal to the mitral valve |
| MV mean PG | Trace the velocity envelope of the flow distal to the mitral valve |
| NALC | Nurse Activity Level Completed |
| NIV | Non-invasive Ventilation |
| O2 | Oxygen |
| PaO2 | Partial Pressure of Oxygen in Arterial Blood |
| PCO2 | Partial Pressure of Carbon Dioxide |
| PEEP | Positive End Expiratory Pressure |
| pH | Potential of Hydrogen |
| PI max PG | Maximum pressure gradient of the pulmonic insufficiency |
| PI max vel | Maximum velocity of the pulmonic insufficiency |
| PO2 | Partial Pressure of Oxygen |
| PT | Prothrombin Time |
| PTT | Partial Thromboplastin Time |
| PV V2 max | Maximum velocity of the flow distal to the pulmonic valve |
| RBC | Red Blood Cell Count |
| RDW | Red Cell Distribution Width |
| RNN | Recurrent Neural Network |
| RV-0002 | Dilated RV |
| RV-0016 | Normal right ventricular systolic function |
| RV-0021 | Normal right ventricular wall motion |
| RV-0056 | Normal RV/LV and septum |
| RV-0060 | Right ventricle free wall hypertrophy, _ |
| RV-0065 | Normal right and left ventricular systolic function |
| RV-0068 | Right ventricular wall motion is depressed |
| RV-0069 | Decreased right ventricular systolic function |
| TC02 | Total Carbon Dioxide |
| TSH | Thyroid Stimulating Hormone |
| TV A max level | A-point maximum velocity of the tricuspid flow |
| TV E max level | E-point maximum velocity of the tricuspid flow |
| TV E/A | (TV E max level) / (TV A max level) |
| TV Lat Peak A’ Vel | Peak A-wave velocity of the left ventricular lateral tricuspid valve annulus tissue doppler spectrum |
| TV Lat Peak E’ Vel | Peak E-wave velocity of the lateral tricuspid valve annulus tissue doppler spectrum |
| TV Lat Peak S’ Vel | Peak S-wave velocity of the lateral tricuspid valve annulus tissue doppler spectrum |
| Tei Index LV (LV MPI) | ((TDI Inter-mitral time LV - TDI ejection time LV) / TDI ejection time LV) |
| VBG | Venous Blood Gas |
| WAT1 | Withdrawal Assessment Tool Score |
