# Supplementary material for: Predicting High Flow Nasal Cannula Failure in an Intensive Care Unit Using a Recurrent Neural Network With Transfer Learning and Input Data Perseveration: Retrospective Analysis
Source: JMIR Med Inform. 2022 Mar 3;10(3):e31760. doi: 10.2196/31760 (PMC8931642; doi:10.2196/31760)
Supplement: Multimedia Appendix 6 [file medinform_v10i3e31760_app6.docx]

**Table A-6** List of the input variables used in the LR-14 model.

| **LR-14 Model Input Variables** |
| --- |
| Heart Rate |
| Mean Arterial Pressure |
| Pulse Oximetry |
| Respiratory Rate |
| Systolic Blood Pressure |
| Weight |
| ABG PCO2 |
| ABG pH |
| CBG PCO2 |
| CBG pH |
| sf_ratio |
| VBG PCO2 |
| VBG pH |
| Age |
