# Supplementary material for: Predicting High Flow Nasal Cannula Failure in an Intensive Care Unit Using a Recurrent Neural Network With Transfer Learning and Input Data Perseveration: Retrospective Analysis
Source: JMIR Med Inform. 2022 Mar 3;10(3):e31760. doi: 10.2196/31760 (PMC8931642; doi:10.2196/31760)
Supplement: Multimedia Appendix 7 [file medinform_v10i3e31760_app7.docx]

**Table A-7.** Number of HFNC trials remaining at various evaluation points.

| **Time on HFNC (hours)** | **Test Set Cohort** | | | **Respiratory Cohort** | | |
| --- | --- | --- | --- | --- | --- | --- |
|  | **Remaining # of Trials: Successful** | **Remaining # of Trials: Failures** | **Total # of Remaining Trials** | **Remaining # of Successes** | **Remaining # of Failures** | **Total # of Remaining Trials** |
| **0.0** | 164 | 42 | 206 | 113 | 28 | 141 |
| **1.0** | 164 | 42 | 206 | 113 | 28 | 141 |
| **2.0** | 164 | 40 | 204 | 113 | 27 | 140 |
| **3.0** | 164 | 38 | 202 | 113 | 26 | 139 |
| **4.0** | 164 | 35 | 199 | 113 | 23 | 136 |
| **5.0** | 164 | 30 | 194 | 113 | 21 | 134 |
| **6.0** | 163 | 29 | 192 | 113 | 21 | 134 |
| **7.0** | 163 | 22 | 185 | 113 | 14 | 127 |
| **8.0** | 163 | 22 | 185 | 113 | 14 | 127 |
| **9.0** | 163 | 19 | 182 | 113 | 11 | 124 |
| **10.0** | 163 | 18 | 181 | 113 | 11 | 124 |
| **11.0** | 162 | 16 | 178 | 112 | 10 | 122 |
| **12.0** | 162 | 14 | 176 | 112 | 9 | 121 |
| **13.0** | 162 | 12 | 174 | 112 | 7 | 119 |
| **14.0** | 162 | 12 | 174 | 112 | 7 | 119 |
| **15.0** | 162 | 11 | 173 | 112 | 7 | 119 |
| **16.0** | 160 | 9 | 169 | 110 | 6 | 116 |
| **17.0** | 160 | 9 | 169 | 110 | 6 | 116 |
| **18.0** | 159 | 9 | 168 | 110 | 6 | 116 |
| **19.0** | 159 | 7 | 166 | 110 | 6 | 115 |
| **20.0** | 159 | 5 | 164 | 110 | 4 | 114 |
| **21.0** | 158 | 2 | 160 | 109 | 2 | 111 |
| **22.0** | 158 | 1 | 159 | 109 | 1 | 110 |

**Table A-8**. Sensitivity vs Specificity of the 2-hour predictions in the entire test set. The highest specificity along each row (fixed sensitivity) is in **bold**.

| **Sensitivity** | **SpecificityLR-14** | **SpecificityLR-517** | **SpecificityLSTM** | **SpecificityLSTM +3xPers** | **SpecificityLSTM+TL** | **SpecificityLSTM +3xPerx +TL** | **Specificity Simple-EN-LSTM+ 3xPers+TL** | **SpecificitySimple-EN-LSTM+ 3xPers+TL** |
| --- | --- | --- | --- | --- | --- | --- | --- | --- |
| 0.10 | 0.98 | 0.98 | **0.99** | **0.99** | 0.98 | 0.98 | 0.98 | 0.98 |
| 0.20 | 0.91 | 0.96 | **0.98** | 0.97 | 0.92 | 0.95 | 0.95 | 0.97 |
| 0.30 | 0.83 | 0.91 | 0.87 | 0.92 | 0.91 | **0.93** | 0.92 | **0.93** |
| 0.40 | 0.73 | 0.87 | 0.80 | **0.89** | 0.87 | 0.88 | **0.89** | 0.85 |
| 0.50 | 0.69 | 0.86 | 0.73 | 0.83 | 0.83 | **0.87** | **0.87** | 0.84 |
| 0.60 | 0.63 | **0.80** | 0.68 | 0.77 | 0.79 | 0.77 | 0.79 | 0.79 |
| 0.70 | 0.63 | 0.68 | 0.54 | 0.57 | 0.74 | 0.74 | 0.73 | **0.79** |
| 0.80 | 0.56 | 0.43 | 0.40 | 0.48 | 0.64 | 0.63 | 0.71 | **0.76** |
| 0.90 | 0.32 | 0.24 | 0.29 | 0.39 | 0.22 | 0.32 | 0.21 | **0.46** |
| 1.00 | 0.05 | 0.03 | 0.16 | **0.18** | 0.04 | 0.05 | 0.04 | 0.05 |

**Table A-9**. Sensitivity vs Positive Predictive Value (PPV) of the 2-hour predictions in the entire test set. The highest PPV along each row (fixed sensitivity) is in **bold**.

| **Sensitivity** | **PPV: LR-14** | **PPV: LR-517** | **PPV: LSTM** | **PPV: LSTM +3xPers** | **PPV: LSTM+TL** | **PPV: LSTM +3xPerx +TL** | **PPV:  Simple-EN-LSTM+ 3xPers+TL** | **PPV: Simple-EN-LSTM+ 3xPers+TL** |
| --- | --- | --- | --- | --- | --- | --- | --- | --- |
| 0.10 | 0.57 | 0.50 | **0.80** | 0.67 | 0.50 | 0.50 | 0.50 | 0.57 |
| 0.20 | 0.35 | 0.53 | **0.67** | 0.62 | 0.38 | 0.50 | 0.50 | 0.62 |
| 0.30 | 0.30 | 0.44 | 0.35 | 0.48 | 0.46 | **0.52** | 0.48 | 0.50 |
| 0.40 | 0.26 | 0.42 | 0.33 | **0.47** | 0.42 | 0.44 | **0.47** | 0.39 |
| 0.50 | 0.28 | 0.47 | 0.31 | 0.42 | 0.42 | **0.48** | **0.48** | 0.43 |
| 0.60 | 0.29 | **0.43** | 0.32 | 0.39 | 0.41 | 0.39 | 0.41 | 0.41 |
| 0.70 | 0.31 | 0.35 | 0.27 | 0.28 | 0.40 | 0.40 | 0.39 | **0.45** |
| 0.80 | 0.31 | 0.26 | 0.25 | 0.27 | 0.35 | 0.34 | 0.40 | **0.45** |
| 0.90 | 0.24 | 0.22 | 0.24 | 0.26 | 0.22 | 0.24 | 0.22 | **0.29** |
| 1.00 | 0.21 | 0.20 | 0.22 | **0.23** | 0.20 | 0.20 | 0.20 | 0.20 |

**Table A-10**. Sensitivity vs Negative Predictive Value (NPV) of the 2-hour predictions in the entire test set.

| **Sensitivity** | **NPV: LR-14** | **NPV: LR-517** | **NPV: LSTM** | **NPV: LSTM +3xPers** | **NPV: LSTM+TL** | **NPV: LSTM +3xPerx +TL** | **NPV:  Simple-EN-LSTM+ 3xPers+TL** | **NPV: Simple-EN-LSTM+ 3xPers+TL** |
| --- | --- | --- | --- | --- | --- | --- | --- | --- |
| 0.10 | 0.82 | 0.82 | 0.82 | 0.82 | 0.82 | 0.82 | 0.82 | 0.82 |
| 0.20 | 0.82 | 0.83 | 0.83 | 0.83 | 0.83 | 0.83 | 0.83 | 0.83 |
| 0.30 | 0.83 | 0.84 | 0.84 | 0.84 | 0.84 | 0.85 | 0.84 | 0.84 |
| 0.40 | 0.83 | 0.86 | 0.85 | 0.86 | 0.86 | 0.86 | 0.86 | 0.85 |
| 0.50 | 0.85 | 0.88 | 0.86 | 0.87 | 0.87 | 0.88 | 0.88 | 0.87 |
| 0.60 | 0.87 | 0.89 | 0.88 | 0.89 | 0.89 | 0.89 | 0.89 | 0.89 |
| 0.70 | 0.90 | 0.90 | 0.88 | 0.89 | 0.91 | 0.91 | 0.91 | 0.92 |
| 0.80 | 0.92 | 0.90 | 0.89 | 0.91 | 0.93 | 0.93 | 0.94 | 0.94 |
| 0.90 | 0.93 | 0.91 | 0.92 | 0.94 | 0.90 | 0.93 | 0.89 | 0.95 |
| 1.00 | 1.00 | 1.00 | 1.00 | 1.00 | 1.00 | 1.00 | 1.00 | 1.00 |
