# Supplementary material for: Predicting High Flow Nasal Cannula Failure in an Intensive Care Unit Using a Recurrent Neural Network With Transfer Learning and Input Data Perseveration: Retrospective Analysis
Source: JMIR Med Inform. 2022 Mar 3;10(3):e31760. doi: 10.2196/31760 (PMC8931642; doi:10.2196/31760)
Supplement: Multimedia Appendix 9 [file medinform_v10i3e31760_app9.docx]

**Table A-9**. Test set AUROCs of the eight models considered at various time points

following HFNC initiation for children with a respiratory diagnosis. The highest AUROC in a column is in bold font.

| **Model** | **0-Hr AUROC** | **0.5-Hr AUROC** | **1-Hr AUROC** | **2-Hr AUROC** | **6-Hr AUROC** | **12-Hr AUROC** |
| --- | --- | --- | --- | --- | --- | --- |
| LR-14 | 0.63 | 0.61 | 0.63 | 0.66 | 0.75 | 0.71 |
| LR-517 | 0.66 | 0.66 | **0.77** | 0.78 | **0.84** | 0.85 |
| LSTM | 0.53 | 0.61 | 0.63 | 0.70 | 0.76 | 0.82 |
| LSTM+3xPers | 0.56 | 0.64 | 0.65 | 0.76 | 0.77 | 0.77 |
| LSTM+TL | 0.71 | 0.69 | 0.69 | 0.74 | 0.81 | 0.86 |
| LSTM+3xPers+TL | 0.66 | 0.65 | 0.70 | 0.76 | 0.81 | 0.88 |
| Simple-EN-LSTM+3xPers+TL | 0.66 | 0.66 | 0.71 | 0.78 | 0.82 | 0.87 |
| Multi-EN-LSTM+3xPers+TL | **0.70** | **0.70** | 0.74 | **0.82** | 0.83 | **0.89** |
