# Supplementary figures and images for: Predicting High Flow Nasal Cannula Failure in an Intensive Care Unit Using a Recurrent Neural Network With Transfer Learning and Input Data Perseveration: Retrospective Analysis
Source: JMIR Med Inform. 2022 Mar 3;10(3):e31760. doi: 10.2196/31760 (PMC8931642; doi:10.2196/31760)

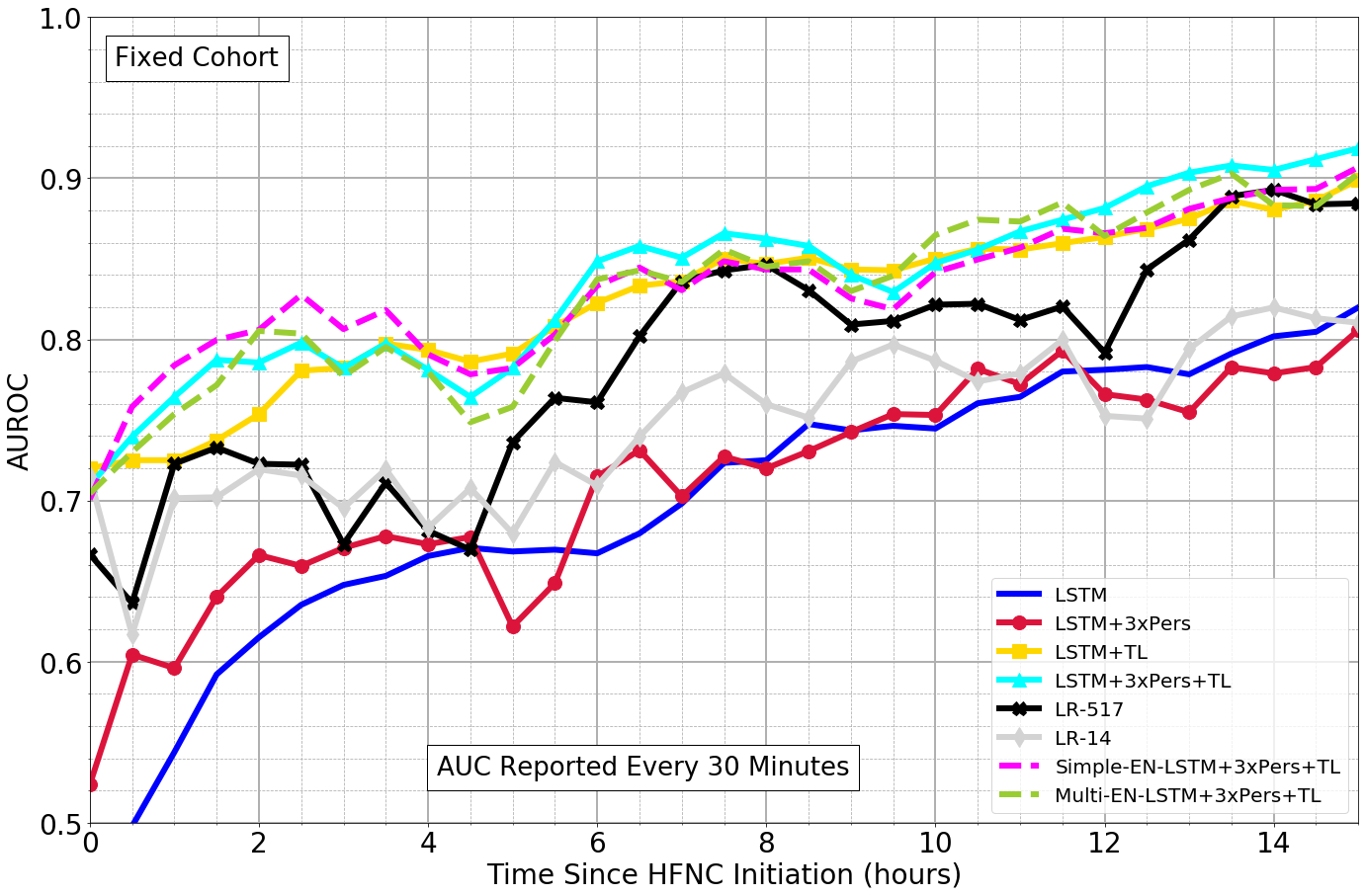

Supplement: Multimedia Appendix 10 [file medinform_v10i3e31760_app10.png]

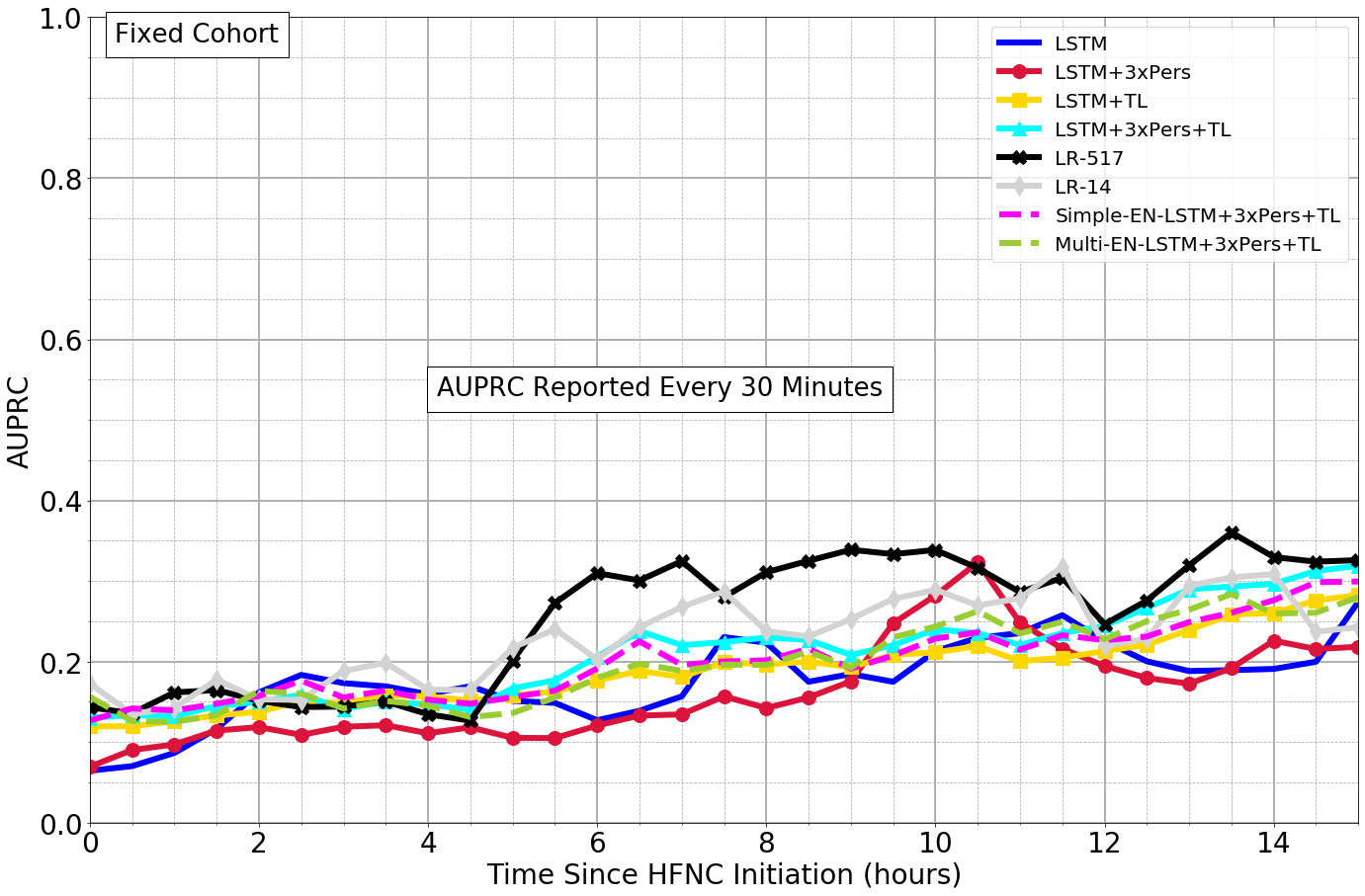

Supplement: Multimedia Appendix 11 [file medinform_v10i3e31760_app11.png]
