# Supplementary material for: Predicting High Flow Nasal Cannula Failure in an Intensive Care Unit Using a Recurrent Neural Network With Transfer Learning and Input Data Perseveration: Retrospective Analysis
Source: JMIR Med Inform. 2022 Mar 3;10(3):e31760. doi: 10.2196/31760 (PMC8931642; doi:10.2196/31760)
Supplement: Multimedia Appendix 12 [file medinform_v10i3e31760_app12.docx]

**Table A-12**. Sensitivity vs Specificity of the 2-hour predictions in the entire test set. The highest specificity along each row (fixed sensitivity) is in **bold**.

| **Sensitivity** | **SpecificityLR-14** | **SpecificityLR-517** | **SpecificityLSTM** | **SpecificityLSTM +3xPers** | **SpecificityLSTM+TL** | **SpecificityLSTM +3xPerx +TL** | **Specificity Simple-EN-LSTM+ 3xPers+TL** | **SpecificityMulti-EN-LSTM+ 3xPers+TL** |
| --- | --- | --- | --- | --- | --- | --- | --- | --- |
| 0.10 | 0.98 | 0.98 | **0.99** | **0.99** | 0.98 | 0.98 | 0.98 | 0.98 |
| 0.20 | 0.91 | 0.96 | **0.98** | 0.97 | 0.92 | 0.95 | 0.95 | 0.97 |
| 0.30 | 0.83 | 0.91 | 0.87 | 0.92 | 0.91 | **0.93** | 0.92 | **0.93** |
| 0.40 | 0.73 | 0.87 | 0.80 | **0.89** | 0.87 | 0.88 | **0.89** | 0.85 |
| 0.50 | 0.69 | 0.86 | 0.73 | 0.83 | 0.83 | **0.87** | **0.87** | 0.84 |
| 0.60 | 0.63 | **0.80** | 0.68 | 0.77 | 0.79 | 0.77 | 0.79 | 0.79 |
| 0.70 | 0.63 | 0.68 | 0.54 | 0.57 | 0.74 | 0.74 | 0.73 | **0.79** |
| 0.80 | 0.56 | 0.43 | 0.40 | 0.48 | 0.64 | 0.63 | 0.71 | **0.76** |
| 0.90 | 0.32 | 0.24 | 0.29 | 0.39 | 0.22 | 0.32 | 0.21 | **0.46** |
| 1.00 | 0.05 | 0.03 | 0.16 | **0.18** | 0.04 | 0.05 | 0.04 | 0.05 |
