# Supplementary material for: Predicting High Flow Nasal Cannula Failure in an Intensive Care Unit Using a Recurrent Neural Network With Transfer Learning and Input Data Perseveration: Retrospective Analysis
Source: JMIR Med Inform. 2022 Mar 3;10(3):e31760. doi: 10.2196/31760 (PMC8931642; doi:10.2196/31760)
Supplement: Multimedia Appendix 13 [file medinform_v10i3e31760_app13.docx]

**Table A-13**. Sensitivity vs Positive Predictive Value (PPV) of the 2-hour predictions in the entire test set. The highest PPV along each row (fixed sensitivity) is in **bold**.

| **Sensitivity** | **PPV: LR-14** | **PPV: LR-517** | **PPV: LSTM** | **PPV: LSTM +3xPers** | **PPV: LSTM+TL** | **PPV: LSTM +3xPerx +TL** | **PPV:  Simple-EN-LSTM+ 3xPers+TL** | **PPV: Multi-EN-LSTM+ 3xPers+TL** |
| --- | --- | --- | --- | --- | --- | --- | --- | --- |
| 0.10 | 0.57 | 0.50 | **0.80** | 0.67 | 0.50 | 0.50 | 0.50 | 0.57 |
| 0.20 | 0.35 | 0.53 | **0.67** | 0.62 | 0.38 | 0.50 | 0.50 | 0.62 |
| 0.30 | 0.30 | 0.44 | 0.35 | 0.48 | 0.46 | **0.52** | 0.48 | 0.50 |
| 0.40 | 0.26 | 0.42 | 0.33 | **0.47** | 0.42 | 0.44 | **0.47** | 0.39 |
| 0.50 | 0.28 | 0.47 | 0.31 | 0.42 | 0.42 | **0.48** | **0.48** | 0.43 |
| 0.60 | 0.29 | **0.43** | 0.32 | 0.39 | 0.41 | 0.39 | 0.41 | 0.41 |
| 0.70 | 0.31 | 0.35 | 0.27 | 0.28 | 0.40 | 0.40 | 0.39 | **0.45** |
| 0.80 | 0.31 | 0.26 | 0.25 | 0.27 | 0.35 | 0.34 | 0.40 | **0.45** |
| 0.90 | 0.24 | 0.22 | 0.24 | 0.26 | 0.22 | 0.24 | 0.22 | **0.29** |
| 1.00 | 0.21 | 0.20 | 0.22 | **0.23** | 0.20 | 0.20 | 0.20 | 0.20 |
