# Supplementary material for: Predicting High Flow Nasal Cannula Failure in an Intensive Care Unit Using a Recurrent Neural Network With Transfer Learning and Input Data Perseveration: Retrospective Analysis
Source: JMIR Med Inform. 2022 Mar 3;10(3):e31760. doi: 10.2196/31760 (PMC8931642; doi:10.2196/31760)
Supplement: Multimedia Appendix 14 [file medinform_v10i3e31760_app14.docx]

**Table A-14**. Sensitivity vs Negative Predictive Value (NPV) of the 2-hour predictions in the entire test set.

| **Sensitivity** | **NPV: LR-14** | **NPV: LR-517** | **NPV: LSTM** | **NPV: LSTM +3xPers** | **NPV: LSTM+TL** | **NPV: LSTM +3xPerx +TL** | **NPV:  Simple-EN-LSTM+ 3xPers+TL** | **NPV: Multi-EN-LSTM+ 3xPers+TL** |
| --- | --- | --- | --- | --- | --- | --- | --- | --- |
| 0.10 | 0.82 | 0.82 | 0.82 | 0.82 | 0.82 | 0.82 | 0.82 | 0.82 |
| 0.20 | 0.82 | 0.83 | 0.83 | 0.83 | 0.83 | 0.83 | 0.83 | 0.83 |
| 0.30 | 0.83 | 0.84 | 0.84 | 0.84 | 0.84 | 0.85 | 0.84 | 0.84 |
| 0.40 | 0.83 | 0.86 | 0.85 | 0.86 | 0.86 | 0.86 | 0.86 | 0.85 |
| 0.50 | 0.85 | 0.88 | 0.86 | 0.87 | 0.87 | 0.88 | 0.88 | 0.87 |
| 0.60 | 0.87 | 0.89 | 0.88 | 0.89 | 0.89 | 0.89 | 0.89 | 0.89 |
| 0.70 | 0.90 | 0.90 | 0.88 | 0.89 | 0.91 | 0.91 | 0.91 | 0.92 |
| 0.80 | 0.92 | 0.90 | 0.89 | 0.91 | 0.93 | 0.93 | 0.94 | 0.94 |
| 0.90 | 0.93 | 0.91 | 0.92 | 0.94 | 0.90 | 0.93 | 0.89 | 0.95 |
| 1.00 | 1.00 | 1.00 | 1.00 | 1.00 | 1.00 | 1.00 | 1.00 | 1.00 |
